# Removing Fractured Endodontic NiTi Instruments with a Tube Technique: Influence of Pre-Treatment with Various Agents on Adhesive Forces In Vitro

**DOI:** 10.3390/ma13010144

**Published:** 2019-12-30

**Authors:** Sebastian Bürklein, David Donnermeyer, Michael Wefelmeier, Edgar Schäfer, Kent Urban

**Affiliations:** 1 Central Interdisciplinary Ambulance in the School of Dentistry, University of Münster, Albert-Schweitzer-Campus 1/W30, 48149 Münster, Germany; eschaef@uni-muenster.de; 2 Department of Operative Dentistry, University of Münster, Albert-Schweitzer-Campus 1/W30, 48149 Münster, Germany; david.donnermeyer@ukmuenster.de; 3 Private Office, Rosenplatz 10, 48143 Münster, Germany; m.wefel@outlook.de; 4 Private Office, Kieferchirurgie Gera, Lessingstraße 2, 07545 Gera, Germany; kent-urban@gmx.de

**Keywords:** adhesion, bonding, luting, nickel–titanium, tube technique

## Abstract

The aim of the study was to evaluate the adhesive forces for removing iatrogenically fractured endodontic nickel-titanium instruments using a modified tube technique with various pre-treatment agents in combination with a light-curing composite. 120 Nickel-Titanium-Mtwo instruments were cut at its parallel shaft and fixed in a vise with an overlap of 2 mm. The surfaces were treated with different agents: A) GC Metalprimer; B) Prime and Bond active; C) NaOCl (3%); D) citric acid (15%); E) phosphoric acid (37%) and group (F) was not pretreated (control). One end of a matching microtube, filled with light-curing composite, was placed over the instrument and a transmitting glass fiber inserted from the opposite side guaranteed polymerization. Pull-out tests (1 mm/min) were performed and failure load was measured digitally. Data were statistically analyzed using the ANOVA and Student–Newman–Keuls tests. Interfaces were subjected to SEM analysis. Prime and Bond active created significant higher pull-out values (mean 30.5 N) compared to all other groups (*p* < 0.001) and Metalprimer (18.5 N) was significantly superior to the untreated (12.6 N) and NaOCl (11.7 N) group (*p* < 0.05). No significant differences were obtained between the other groups (*p* > 0.05). Thus, adhesives improved bonding to fractured NiTi instruments.

## 1. Introduction

Chemo-mechanical disinfection of the entire root canal system is crucial for successful root canal treatment [1]. During preparation, endodontic instruments may fracture due to torsional load or cyclic fatigue with an incidence ranging between 1.8% and 3.3% [2,3]. This accidental mishap is undesired and may negatively influence treatment outcome as the fragment may block root canal areas making them inaccessible for both mechanical and chemical disinfection [4,5,6], depending on the stage of root canal-procedure when fracture occurred [3,7,8,9]. Hence, in some clinical scenarios the removal of the fractured instruments is indicated [10].

All efforts in managing this undesired complication should be based on a thorough knowledge of each treatment option considering the success rates and should be well balanced against potential risks of leaving or removal of the fragment [8]. It has to be taken into consideration that fractured endodontic instruments might not directly affect the prognosis of the tooth [3,5,9,11], because fragments made of stainless steel or nickel–titanium (NiTi) itself may not directly lead to infection.

For clinicians several nonsurgical treatment options are available: Besides the “braiding technique” [12], where small files are used in order to remove or at least bypass the instrument, the use of ultrasonic devices is an effective and commonly used way to expose and eventually remove fragments [13,14]. If ultrasonic procedures fail, the tube technique is another suitable option to remove fractured instruments [14].

The oldest system, the Masserann-Kit (Micro-Mega, Besançon, France) [15] is associated with a considerable weakening of root dentin and an increased risk of perforation due to the use of large and rigid trephine drills [16,17]. A more gentle and tooth structure conserving approach represents the IRS (instrument removal system; San Diego Swiss Machining, San Diego, CA, USA) that only needs a straight line access of 0.6 mm in diameter. However, the fractured instrument needs to be exposed at least up to 2–3 mm [14]. Alternatively, a microtube filled with superglue (cyanoacrylate) or with dual-curing composite can be shifted over the exposed end of the fractured instrument [18]. Using microtubes filled with adhesive materials or auto-polymerization composites is associated with some disadvantages when compared to mechanical systems, e.g., the extended superglue may set outside the tube and inside the root canal and adhere to the dentin walls. Thus, cleaning of the entire root canal system needed after failed removal trials may be time consuming [14]. Setting of the material is less predictable, sometimes tedious and may be incomplete resulting in unsuccessful retrievals of the instruments. Additionally, only relatively low tensile forces were achieved [14]. Wire-loop techniques are further options for retrieval of fractured fragments [19,20].

Recently, light curing composite was proposed for fixation of the fractured instrument [21]. In a first step a tube with an inner diameter close to the cross sectional diameter of the fractured instrument is filled with a light-curing composite and placed over the bared portion of the fragment. In a second step light curing is achieved by placing a glass fiber from the backside into the tube until it gets into contact with the end of the fragment. After setting of the composite, high forces can be transmitted to the “instrument-tube-complex” consisting of the following components: fractured instrument, composite and tube. The major advantages are the minor necessary baring of the broken instrument compared to other methods and the easy cleaning of the root canals because material only cures in the areas where the light is emitted [21]. Exposure of the fragment of 1 mm seems to be enough to achieve sufficient adhesion and to remove the fragment [21]. The smallest available glass-fiber is 0.25 mm—meaning the inner diameter of the used tube has to be at least 0.26 mm. 

First investigations assessing the tube technique using light curing composite were limited to stainless steel endodontic instruments [21]. It is generally perceived that NiTi-instruments are more prone to fracture than their stainless steel counterparts [22]. However, if NiTi files are used appropriately, the fracture incidence appears to be comparable [22]. Preliminary studies showed that adhesive forces were less than one third of those when using stainless steel instruments. Hence, the aim of the present study was to investigate the influence of different endodontic irrigation solutions (NaOCl and citric acid) and special agents associated with restorative procedures (phosphoric acid, Prime and Bond active and Metalprimer) on adhesive force of NiTi instruments. 

The null-hypothesis tested was that that both irrigation and bonding agents affect adhesion when removing broken instruments using a tube technique with light curing composite. On the one hand, endodontic irrigation solutions (NaOCl and citric acid) and phosphoric acid used for etching procedure prior to adhesive dental fillings may have no effect on adhesive forces to NiTi endodontic instruments and on the other hand, adhesives may improve bonding.

## 2. Materials and Methods

Power analysis (G*Power, Version 3.1.9.2; Heinrich-Heine University of Düsseldorf, Düsseldorf, Germany) was performed by post-hoc-power analysis after obtaining results in order to find out if the sample size was adequately chosen. Overall effect size calculated from means and variances of the data was 1.026. Hence, the used sample size with *n* = 20 per group (α = 0.05) revealed a power of 1.0.

A total of 120 NiTi Mtwo (25/.06) instruments (VDW, Munich, Germany) were cut at their parallel nickel-titanium shaft 2 mm above the 16 mm working part. The outer diameter was 1.2 mm and surfaces were inspected with an operating microscope (OPMI pico, Carl Zeiss, Jena, Germany) under 30-fold magnification to guarantee that no undersized areas or any defects that may influence the adhesive bond were obvious. The instruments were randomly allocated into six groups (*n* = 20 per group) and fixed in a chuck with an overlap of 2 mm. Rubber stoppers prevented any materials used from entering undercut areas of the working part. Surfaces were treated with the following agents:

A) GC Metalprimer (GC, Tokyo, Japan);

B) Prime and Bond active (Dentsply Sirona, Bensheim, Germany);

C) NaOCl (3%);

D) Citric acid (15%);

E) Phosphoric acid (37%);

F) Untreated = control.

All agents were used in accordance to their common use in dental treatment procedures. GC Metalprimer was applied and left to dry. Prime and Bond active was brushed and slightly agitated with an applicator tip for 20 s. Sodium hypochlorite (NaOCl) was brushed for two minutes and citric acid for one minute, respectively–corresponding to regular contact/application times during endodontic irrigation procedure. A replacement/refreshment of the solutions every 30 s guaranteed effectiveness. Phosphoric acid had an exposure time of 30 s as used for etching prior to adhesive restorations.

**Figure 1:**
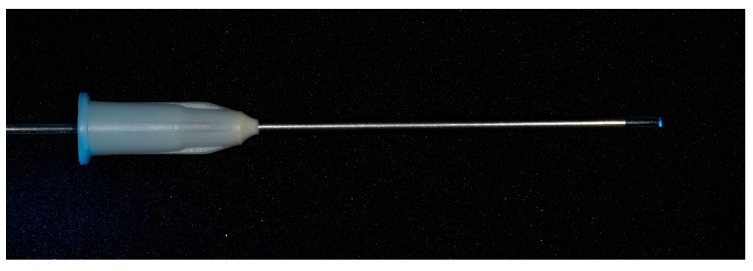
Emitted light through the tube with the glass-fiber.

**Figure 2:**
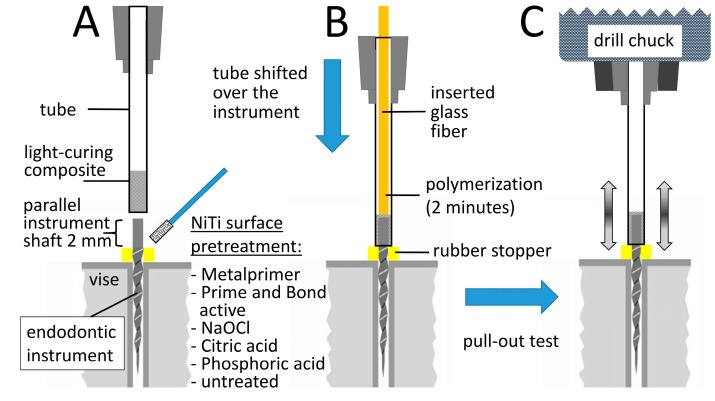
Scheme of the experimental setup with the different steps during the investigation. (**A**) Pretreatment of the clamped instrument and filling of the tube with light-curing SDR composite; (**B**) polymerization procedure after shifting the tube over the instrument and glass fiber insertion from the opposite side and (**C**) pull-out test for failure load of the instrument-composite-tube compound in a universal tensile testing machine.

The light curing composite SDR (Surefil SDR, Dentsply, York, PA, USA) was placed into suction cannulas (Transcodent LL16G, Transcodent, Kiel, Germany) with an inner diameter of 1.3 mm and tips were placed in a standardized approach over the instruments (Figure 1 and Figure 2). From the opposite side of the tube a glass fiber (Conrad Electronic SE, Hirschau, Germany) (Figure 1) with a diameter of 1 mm was inserted, pushed forward until reaching contact with the fragment and composite was light cured for 2 min with 1000 W/cm^2^ (Smartlite PS, Dentsply, York, PA, USA; Figure 1 and Figure 2).

Pull-out tests were performed axially with a constant speed of 1 mm/min using a universal tensile testing machine (Testometric M350-5CT, Rochdale, UK; Figure 2); failure load in Newton was measured digitally.

In addition to the tensile tests, further three specimens were prepared in each group and subjected to a scanning electron microscopic examination (SEM; Zeiss, EVO MA10; Carl Zeiss, Jena, Germany) for analysis of the interfaces of the involved components. Prior to examination samples were embedded in Technovit 4071 (Heraeus Kulzer, Wehrheim, Germany), cut longitudinally, ground to a 4000-grit size and polished. 

Data were distributed normally (Kolmogorov–Smirnov and Shapiro–Wilk test) and statistically analyzed using ANOVA and Student-Newman-Keuls tests using the Statview program (SAS institute, Cary, NC, USA). The level of significance was set at *p* < 0.05.

## 3. Results

**Figure 3:**
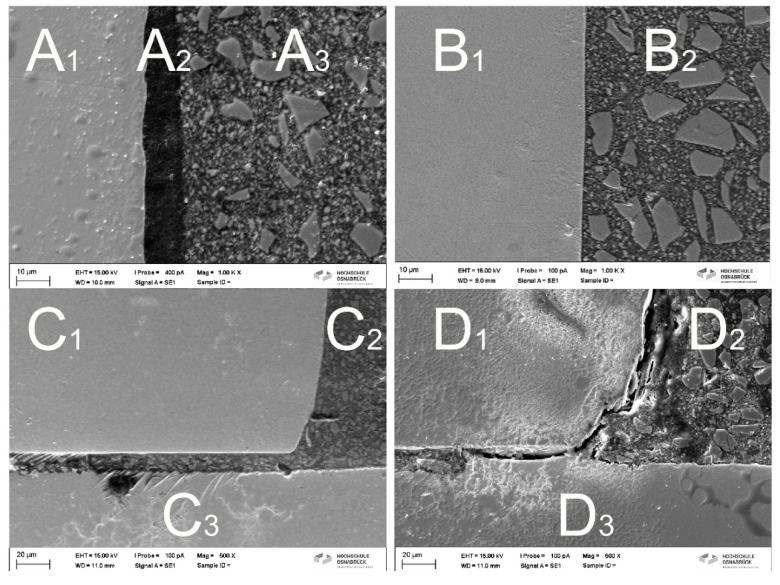
Interfaces of the different groups: (**A**) gapless interface between A1: NiTi instrument A2: Prime and Bond active and A3: SDR composite (original magnification ×1000) and (**B**) gapless interface between B1: NiTi instrument and B2: SDR composite with the use of Metalprimer (original magnification ×1000). (**C**) Gap-free interface between C1: instrument C2: composite: and C3: suction cannula (original magnification ×500) with the use of Metalprimer and (**D**) interface between D1: NiTi-instrument D2: composite and D3 tube (untreated instrument; original magnification ×500) with marked gaps.

The data are listed in Table 1. Exemplary longitudinal sections can be seen in Figure 3. Prime and Bond active (Figure 3A) was associated with significantly greater pull-out values compared to all other groups (*p* < 0.001). Pull-out values of Metalprimer (Figure 3B,C) were significantly higher compared to the untreated (Figure 3D) and NaOCl (11.7 N) group (*p* < 0.05). No significant differences were obtained between all other groups (*p* > 0.05).

Only adhesive failure modes at the interface between the NiTi instrument and the composite resin were detected in all groups.

The exemplary longitudinal sections evaluated by SEM (Figure 3) highlighted the different behavior of the materials used and their impact on adhesion. Whereas Metalprimer and Prime and Bond active created gapless interfaces in the close-up views (Figure 3A,B) and also in the overview image with lower magnification showing half of the adhesive joint (e.g., Metalprimer group; Figure 3C), all other groups (e.g., untreated group; Figure 3D) were associated with obvious gaps, offering less adhesive surface.

## 4. Discussion

The main problem in loosening fractured intracanal NiTi instruments is the super-elastic behavior of the NiTi alloy (=SE NiTi) that may damp the vibrations applied by sonic or ultrasonic tips and fragments with a length of more than 4.5 mm are described being almost irretrievable using ultrasonic equipment alone [23,24]. The currently available more flexible thermally modified NiTi alloys that offer controlled memory effects (e.g., CM-wire, Blue-wire, Gold-wire and Max-wire) could further enhance this effect. Additionally, exposed portions of the instrument fragment may fracture again when using ultrasonic tips, thereby further impeding the removal attempt. Therefore, the tube technique may be a promising alternative from a clinical point of view to remove the fractured instruments after exposure.

The authors are well aware of the fact that this pilot in vitro study investigated non-evidence based concepts of instrument fracture removal. Moreover, some adhesive/bonding agents were used beyond the recommendations of the manufacturers. However, the main attempt was to assess if, from a basic science point of view, different agents may have the potential to improve adhesive forces when using a tube technique. At the present moment, the findings of this study do not justify to transfer this concept to the routine dental practice. Before, several aspects require further in-depth investigations, as particularly the safety of the procedure (blocking of the root canal with adhesives, penetration of ingredients of the adhesive into the periradicular tissues). Moreover, the approved area of application of some adhesives must be extended by the manufacturer before the present findings can be adopted as a clinical concept.

The experimental setup was configured in a way that the pull-out forces were measured using the parallel shafts of the NiTi instruments in order to evaluate the pure interaction between the interface agents/solutions and the instrument surface. Endodontic instruments possess cutting edges and undercut areas that cause relatively high pull-out forces [21] and therefore the measured pull-out values would be considerably influenced by the geometrical shape of the working part of the instruments.

NiTi alloys show a passivation layer of their surface. This surface layer consists mainly of a titanium-oxide layer (TiO_2_) [25] and increases its stability by creating a chemical and physical barrier against oxidation of nickel and leading to a corrosion protection of the material [26]. The passivating layer of NiTi alloys may hamper the adhesive compound. Hence, pretreating the NiTi surfaces may have beneficial aspects [27]. Therefore, the experimental groups were selected according to commonly used endodontic irrigation protocols (NaOCl and citric acid) and different agents related to adhesive restorations (pretreatment: phosphoric acid; bonding: Prime and Bond active) or prosthodontic restorations (Metalprimer). The untreated group served as a control. Setup based on dry conditions due to the uniformity of testing and the usually acceptable drying possibilities after exposure of the fractured instruments even under clinical conditions. The primary focus was on the impact of the agents on the adhesive force without the influence of additional parameters—especially, without humidity/moisture as a poorly reliable factor.

The null-hypothesis was accepted as both irrigation and bonding agents affected adhesion and consequently pull-out forces. The significantly highest pull-out forces were obtained when using light-cured composite after pretreatment with Prime and Bond active (*p* < 0.05; Table 1). Sodium hypochlorite as the main irrigant during root canal treatment had no impact on the pull-out force as no significant difference in comparison with the untreated control was obtained (*p* > 0.05). In addition, citric acid (as a representative for smear layer removal in endodontic irrigation protocols) and phosphoric acid (representative for an etching agent in adhesive dentistry) did not improve pull-out forces significantly compared to the untreated group (*p* > 0.05). Hence, regular irrigation solutions (NaOCl and citric acid) and acids (phosphoric acid) were unsuited to improve pull-out forces. Practitioners should be aware that endodontic irrigation solutions do not positively affect adhesion. Nevertheless, during endodontic treatment irrigation is crucial. When broken instruments have to be removed, those irrigants should be washed out before using the luting technique with light curing composite. Prime and Bond active or Metalprimer may be useful agents to improve the adhesion to NiTi instruments.

Metalprimer directly affects surfaces of alloys by creating reactive molecules (Figure 3B,C) allowing a chemical bond between the composite and the NiTi alloy [28]. In general, Metalprimers contain VBATDT (5-(4-vinylbenzyl)-2-thiobarbituric acid (5VS), 6-(4-vinylbenzyl-n-propyl) amino-1,3,5-triazine-2,4-dithione), MDP (10-Methacryloyloxydecyl Dihydrogen Phosphate) and/or MMA (Methyl methacrylate). These ingredients guarantee improved bond strengths to both precious and non-precious metals. The coupling mechanism is based on the following aspects: i) the transformation of thione (–C=S) to thiol (–C–S–H) groups on the metal surface (M) then leading to a primary bond formation (–C–S–M) and (ii) the co-polymerization of vinyl groups with the methacrylate-based resin monomer [28]. Additionally, Metalprimer even improve adherence of resins to NiTi alloys [27,29].

In contrast to the chemical bond caused by the Metalprimer, Prime and Bond active has an extremely low surface tension (viscosity < 30 mPa*s) so that this adhesive spreads out into the thinnest pores and depressions of the NiTi surface, resulting in increased microtensile bond strengths [30]. Additionally, the one-bottle adhesive Prime and Bond active also contains 10-methacryloyloxydecyl dihydrogen phosphate (= MDP). This component is identical to a Metalprimer ingredient and is claimed to create self-organizing nanolayer structures that enable a water-stable interface with lower residual moisture susceptibility [31]. The SEM figures clearly demonstrated the differences at the interface between the different agents used (Figure 3A–D). While no gaps are visible in the Metalprimer group between the NiTi-instrument and the SDR composite (Figure 3B,C) and between the NiTi-instrument and Prime and Bond active and the SDR composite in the “Prime and Bond active”-group (Figure 3A), distinct gaps are evident at the interface in all other groups (e.g., Figure 3D). Obviously, contact area seems to have an impact on adhesion, too.

## 5. Conclusions

In the present in vitro study, Prime and Bond active significantly increased adhesion of a light curing composite to NiTi instruments when using a modified tube technique. Even in clinical practice, this technique may represent a promising and reliable alternative or at least a useful addition to the existing procedures in removing accidental fractured instruments without excessive weakening of the root canal. Additional factors affecting pull-out forces (e.g., polymerization time and mechanical manipulation due to exposure procedures using ultrasonic instruments) should be investigated in further studies.

## Figures and Tables

**Table 1:** Pull-out forces (values in Newton) achieved with the different pretreatment agents/solutions. Values with the same superscript letters were not statistically different at a significance level *p* < 0.05.

Pull-Out Force [N]	GC Metalprimer	Bonding Prime and Bond Active	Sodium Hypochlorite 3%	Citric Acid 15%	Phosphoric Acid 37%	Untreated
**median**	18.40	31.00	12.15	15.05	14.40	12.40
**mean**	18.53 ^b^	30.54 ^a^	11.70 ^c^	15.21 ^b,c^	14.19 ^b,c^	12.62 ^c^
**sd**	5.45	8.60	4.59	4.95	6.90	5.48
**min**	8.80	17.70	4.10	7.60	7.50	5.90
**max**	31.20	49.50	20.50	25.30	33.80	24.90

^a,b,c^ The superscript letters show the significant differences. Different letters mean “statistically different” with the given significance level; same letters mean “not statistically different” at a significance level *p* < 0.05.
